# BME Estimation of Residential Exposure to Ambient PM_10_ and Ozone at Multiple Time Scales

**DOI:** 10.1289/ehp.0800089

**Published:** 2008-12-15

**Authors:** Hwa-Lung Yu, Jiu-Chiuan Chen, George Christakos, Michael Jerrett

**Affiliations:** 1 Department of Bioenvironmental Systems Engineering, National Taiwan University, Taipei, Taiwan;; 2 Department of Epidemiology, University of North Carolina at Chapel Hill, Chapel Hill, North Carolina, USA;; 3 Department of Geography, San Diego State University, San Diego, California, USA;; 4 Department of Environmental Health, University of California at Berkeley, Berkeley, California, USA

**Keywords:** Bayesian, BME, environment, exposure, spatiotemporal, stochastic

## Abstract

**Background:**

Long-term human exposure to ambient pollutants can be an important contributing or etiologic factor of many chronic diseases. Spatiotemporal estimation (mapping) of long-term exposure at residential areas based on field observations recorded in the U.S. Environmental Protection Agency’s Air Quality System often suffer from missing data issues due to the scarce monitoring network across space and the inconsistent recording periods at different monitors.

**Objective:**

We developed and compared two upscaling methods: UM1 (data aggregation followed by exposure estimation) and UM2 (exposure estimation followed by data aggregation) for the long-term PM_10_ (particulate matter with aerodynamic diameter ≤ 10 μm) and ozone exposure estimations and applied them in multiple time scales to estimate PM and ozone exposures for the residential areas of the Health Effects of Air Pollution on Lupus (HEAPL) study.

**Method:**

We used Bayesian maximum entropy (BME) analysis for the two upscaling methods. We performed spatiotemporal cross-validations at multiple time scales by UM1 and UM2 to assess the estimation accuracy across space and time.

**Results:**

Compared with the kriging method, the integration of soft information by the BME method can effectively increase the estimation accuracy for both pollutants. The spatiotemporal distributions of estimation errors from UM1 and UM2 were similar. The cross-validation results indicated that UM2 is generally better than UM1 in exposure estimations at multiple time scales in terms of predictive accuracy and lack of bias. For yearly PM_10_ estimations, both approaches have comparable performance, but the implementation of UM1 is associated with much lower computation burden.

**Conclusion:**

BME-based upscaling methods UM1 and UM2 can assimilate core and site-specific knowledge bases of different formats for long-term exposure estimation. This study shows that UM1 can perform reasonably well when the aggregation process does not alter the spatiotemporal structure of the original data set; otherwise, UM2 is preferable.

Many human exposure and epidemiologic studies have investigated associations between pollutant exposure and disease risk and their potential consequences (Aickin 2002; Chen et al. 2004). For studies on ambient air pollutants, because of the substantial cost and logistic constraints, personal exposure monitoring can be used only for a small number of study participants, thus resulting in low statistical power to detect small effects (Ozkaynak et al. 1996). Most air pollution epidemiologic investigations use individual health data sets at nationwide or regional scales to assess the subtle risks of pollution exposure. In these cases, ambient air-quality monitoring networks, such as the Air Quality System (AQS) operated by the U.S. Environmental Protection Agency (EPA), constitute important and useful environmental data sources concerning the acute and chronic effects of ambient pollutants (U.S. EPA 2002).

Although these environmental monitoring data sources provide useful information to estimate human exposure across space and time, environmental epidemiologists and exposure scientists still face several practical and methodologic challenges in analyzing and modeling the environmental data (Li et al. 2008; Mutshinda et al. 2008).

One challenge is the geographic coverage of the region of interest. Ideally, if the pollution-monitoring stations are located near the residences of the study participants, a participant’s exposure could be easily estimated from neighboring pollutant observations (Maxwell and Kastenberg 1999; Wu et al. 2004). Unfortunately, the AQS monitoring network is relatively scarce compared with the number and geographic distribution of participants considered in large epidemiologic studies, and the geographic locations with direct ambient observations are often at large distances from the places where the study participants reside. To address this issue while assessing individual-level exposures, the geocoding of the subjects’ residential addresses is usually combined with some form of interpolation of likely pollution levels between monitoring locations. Spatial interpolation techniques can be used to estimate large-region pollutant exposures, including deterministic inverse distance schemes (Michelozzi et al. 2002), Monte Carlo methods (Kentel and Aral 2005), and kriging techniques (Christakos and Thesing 1993; Liao et al. 2006; Rushton et al. 1996). Kriging techniques, in particular, have been applied with increasing frequency in large-scale epidemiologic studies, including long-term exposure assessment (Brauer et al. 2003; Hoek et al. 2001). However, because of their inherent constraints (estimator linearity, probabilistic normality, and limited interpretive features that cannot consider highly relevant qualitative knowledge), the mainstream kriging techniques do not always address successfully important human exposure issues, including the integration of composite space–time dependencies and the assimilation of soft (uncertain) information sources that are prevalent in most human exposure studies.

The second issue relates to the limited sampling frequency of environmental monitoring networks. For example, the current AQS monitoring database includes particulate matter (PM) data sampled at 1-, 2-, 3-, 6-, and 9-day cycles (note that most data are sampled at a 6-day cycle). As a result, even if the residences of the study participants are very close to ambient air monitoring stations, some considerable pollution events likely occur during times when the local monitoring stations are not operating. To address this issue, which is often a significant concern to time-series analyses and epidemiologic panel studies of acute health effects, a smoothing technique is often applied to estimate ambient air pollutant levels that were missing during the times of interest (Conceicao et al. 2001; Sagiv et al. 2005). Nevertheless, neither spatial nor temporal analyses have fully accounted for and taken advantage of the exposure variability generated in a composite space–time dependence domain. Remarkably, the temporal domain of AQS air pollution monitoring is considerably more extensive than its spatial domain. This suggests that, especially in studies where exposures at multiple time scales need to be estimated, extending purely spatial or purely temporal interpolation techniques in a composite space–time context would improve considerably the quality of the information used in exposure estimation (Wang et al. 2008). Not surprisingly, several case studies have explicitly demonstrated that ignoring space–time cross-effects can lead to larger errors in pollution estimation (Christakos and Serre 2000; Christakos and Vyas 1998; Christakos et al. 2001; Vyas and Christakos 1997).

The third major issue is how to aggregate data and estimate exposure at time scales that are relevant to the study outcome. To study acute effects, the exposure is often assessed at small time scales (e.g., hourly or daily) (Stallard and Whitehead 1995; Tamborini et al. 1990). In chronic disease studies, such as lung cancer or cardiovascular diseases, average exposures at large time scales (e.g., monthly or yearly) are often used to represent the cumulative long-term exposures (Katsouyanni and Pershagen 1997; Nyberg et al. 2000). A desirable feature in defining the exposure time scale is to align or reference the estimated exposure values to the timing of the study outcome, because such an approach allows epidemiologists to explore the temporal relationships between index exposure and event occurrence, while accounting for the presence of induction time or latency period. To achieve this goal in large time-scale pollution estimation, aggregation of exposure data at small time scales is needed because daily exposure information is not always available from the existing air monitoring networks. Then, one may first aggregate the environmental monitoring data at small time scales and then apply an interpolation technique to estimate exposure at the large time scale of interest. Alternatively, one may first interpolate the individual-level exposure (e.g., residential exposure) at small time scales, followed by the aggregation of all estimated exposure values from small time scales to derive exposure values at large time scales. Because the existing environmental data with aggregated yearly exposure from air monitoring network are only indexed to calendar years, both approaches offer the advantages of avoiding misalignment between estimated exposures at large time scales and the occurrence of study outcomes. They may also be appealing to researchers interested in differentiating the acute health effects from those related to long-term exposures. Remarkably, the relative performance of these two approaches in the upscaling of environmental exposure data used in health and epidemiologic studies has not been evaluated and compared.

In view of the above considerations, in this study we evaluated and compared the relative performance of two upscaling methods in the analysis and estimation of environmental exposure data at multiple time scales. We also compare the two approaches in the spatio temporal estimation of long-term exposure to ambient air pollutants in the context of the HEAPL (Health Effects of Air Pollution on Lupus) study. In particular, we considered exposures to PM_10_ (PM with aerodynamic diameter ≤ 10 μm) and ozone ambient concentrations. We used the spatiotemporal Bayesian maximum entropy (BME) reasoning and quantitative techniques (Christakos et al. 2005), because they account for the aforementioned issues of individual-level exposure estimation in a mathematically rigorous and interpretively meaningful manner. Numerical implementation of BME in real-world applications is made possible by means of the publicly available SEKS-GUI (Spatiotemporal Epistematics Knowledge Synthesis Model—Graphic User Interface) computer software library (Yu et al. 2007; Kolovos et al. 2006). This software library (SEKS-GUI 2007) was used to analyze the extant AQS data sets in the present study and to derive PM_10_ and ozone exposure estimates across space–time.

## Methods

### Air pollution data processing

The residential locations of the HEAPL study participants are in the Carolinas (states of North and South Carolina), and the time period considered in this analysis is 1995–2002. We obtained PM_10_ and ozone observations for this time period and geographic locations from the AQS database. Each of the raw AQS data sets provides information about the spatial coordinates, collection time, sampling duration, sampling frequency, and data duplication indicators (U.S. EPA 2005).

**Figure 1:**
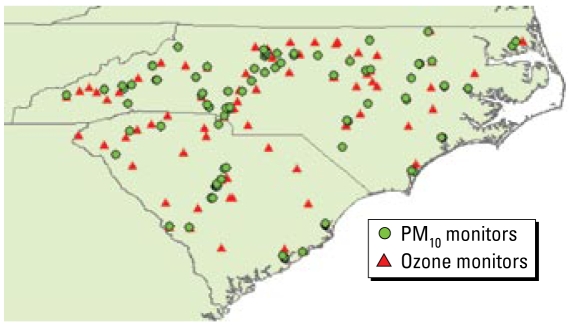
Geographic locations of air pollution monitoring stations.

The PM_10_ (micrograms per cubic meter) and ozone (parts per billion) databases in the study region contained nonuniform data formats and data collection times. A total of 87 PM_10_ monitoring stations were available during the specified study period (1995–2002). Among them, 75 stations generated observations in terms of 24-hr averages every 6 days, whereas the remaining stations recorded hourly; however, only 15 out of 75 daily and 6 out of 12 hourly monitoring stations were in constant operation during the entire study period. In contrast, all of the 77 ozone monitoring stations obtained hourly observations, but only 11 stations operated constantly throughout the study period. Figure 1 shows the spatial distribution of the monitoring stations for both pollutants (PM_10_ and ozone) and the geographic locations of the residences of the study participants.

### Residential data source

**Figure 2:**
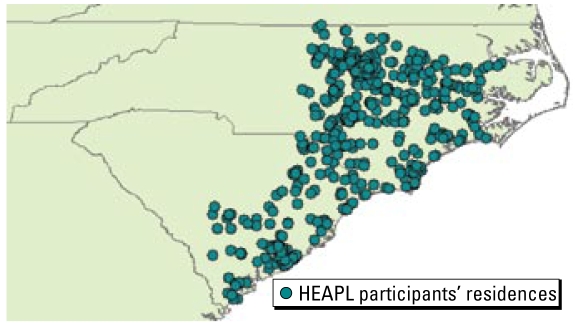
Geographic locations of HEAPL participants’ residences.

HEAPL used extant residential data collected from 620 participants in the Carolina Lupus Study (Cooper et al. 2002). We collected the residential data used in present analyses from the baseline interview that took place in early 1997 to mid-1998 as well as the subsequent interview in 2001. Most participants lived in the eastern and central part of the Carolinas, as shown in Figure 2. To obtain the coordinates (longitudes and latitudes), the geocoding of all study participants’ residential addresses during this period was processed by a specialist at the Cecil G. Sheps Center for Health Services Research at University of North Carolina at Chapel Hill following the standard procedure (Bonner et al. 2003; Ward et al. 2005). The HEAPL study protocols have been approved by the Institutional Review Board of the University of North Carolina at Chapel Hill.

### BME analysis

The BME theory was introduced in geostatistics and space–time statistics by Christakos (2000). BME was later considered in a general epistematics context and applied in the solution of real-world problems in environmental health fields (Choi et al. 2003; Christakos 2009; Law et al. 2006; Savelieva et al. 2005; Serre et al. 2003). BME analysis can incorporate nonlinear exposure estimators and non-Gaussian probability laws, and it can integrate core knowledge (epidemiologic laws, scientific models, theoretical space–time dependence models, etc.) with multisourced, site-specific information at various scales (including aggregated variables and empirical relationships). Central elements of the BME method are described below.

A human exposure attribute (e.g., pollutant concentration) is represented as a spatiotemporal random field (RF) *X****p*** = *X****s****_t_* (Christakos and Hristopulos 1998), where the vector ***p*** = (***s***, *t*) denotes a spatiotemporal point (***s*** is the geographic location and *t* is the time). The RF model is viewed as the collection of all physically possible realizations of the exposure attribute we seek to represent mathematically. It offers a general and mathematically rigorous framework to investigate human exposure that enhances predictive capability in a composite space–time domain. The RF model is fully characterized by its probability density function (pdf) ƒ*_KB_*, which is defined as


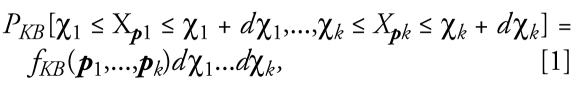


where the subscript KB denotes the “knowledge base” used to construct the pdf.

We considered two major knowledge bases: the core (or general) knowledge base, denoted by G-KB, which includes physical and biological laws, primitive equations, scientific theories, and theoretical models of space–time dependence; and the specificatory (or site-specific) knowledge base, S-KB, which includes exact numerical values (hard data) across space–time, intervals (of possible values), and probability functions (e.g., the datum at the specified location has the form of a probability distribution). The total knowledge base is denoted by *K* = *G* ∪ *S*; that is, it includes both the core and the site-specific knowledge bases.

The fundamental BME equation is as follows (for technical details, see Christakos et al. 2005):


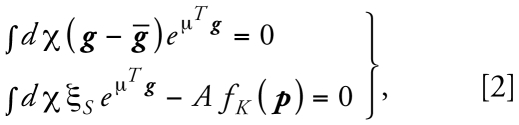


where ***g*** is a vector of *g*_α_ functions (α = 1, 2, …) that represents stochastically the G-KB under consideration (the bar denotes statistical expectation), **μ** is a vector of μ_α_-coefficients that depends on the space–time coordinates and is associated with ***g*** (i.e., μ_α_ expresses the relative significance of each *g*_α_ function in the composite solution sought), ξ*_S_* represents the S-KB available, *A* is a normalization parameter, and ƒ*_K_* is the pollutant or exposure pdf at each space–time point (the subscript *K* means that ƒ*_K_* is based on the total knowledge base that is the blending of the core and site-specific knowledge bases). The vectors ***g*** and ξ*_S_* are inputs in Equation 2, whereas the unknowns are **μ** and ƒ*_K_* across space–time.

The G-KB refers to the entire ***p*** domain of interest, which consists of the space–time point vector ***p****k* where exposure estimates are sought and the point vector ***p***_data_ where site-specific information is available. The G-KB may include theoretical space–time dependence models (mean, covariance, variogram, generalized covariance, multiple-point statistics, and continuity orders) of the exposure attribute represented by the RF *X****p***. Most commonly, however, only the mean and the covariance (or variogram) are used in geostatistics studies of human exposure. In addition, the exposure variables of interest are often log-normally distributed. One cannot avoid noticing that there are serious concerns about the biased estimation of the arithmetic mean on the basis of the log-normal assumption (Parkhurst 1998). In our study, we applied the normal score transformation (Deutsch And Journel 1998) to all PM_10_ and ozone data sets, thus relaxing the log-normal assumption and assuring that the transformed data set is normally distributed.

For practical purposes, the data point vector ***p***_data_ consists of the hard data point vector ***p***_hard_ (where exact measurements are available) and the soft data point vector ***p***_soft_ (where qualitative/incomplete yet valuable information may be available). For illustration, assume that 32 exact PM_10_ observations are available at the space–time points ***p***_hard_ = (***p***_1,_ …, ***p***_32_), that is, *X****p***_1_ = 5.1, …, *X****p***_32_ = 9.3 (in suitable units); and that 55 uncertain PM_10_ data are available at the points ***p***_soft_ = (***p***_33_, …, ***p***_87_), say, of the interval form 3.2 < *X****p***_33_ < 4.1, …, 5.2 < *X****p***_87_ < 6.4 (in suitable units). This sort of site-specific information is mathematically expressed by *P_S_* [*X****p***_1_ = 5.1, …, *X****p***_32_ = 9.3] =1 and *P_S_* [3.2 < *X****p***_33_ < 4.1, …, 5.2 < *X****p***_87_ < 6.4 ] =1, respectively. More generally, assume that at point ***p***_24_ the uncertain datum is expressed by the density function ƒ*_S_* (***p***24); then, *P_S_* [*X****p***_24_ < χ] = ∫_−∞_^χ^ *d*_χ_ *f_S_* (***p***_24_). For several other examples, see Yu et al. (2007).

By incorporating the total K-KB into exposure analysis, the derived pdf ƒ*_K_* in Equation 2 describes the distribution of exposure values at each estimation point ***p****_k_*. Given the ƒ*_K_* at ***p****_k_*, different exposure estimates (most probable, error minimizing, etc., estimates) can be calculated at each spatiotemporal node of the appropriate mapping grid, depending on the objectives of the study. As mentioned above, in this work the BME method is implemented by means of the publicly available SEKS-GUI software library (Kolovos et al. 2006; Yu et al. 2007).

### Multiple time-scale exposure

In the context of HEAPL, we considered air pollution exposure at multiple long time scales (including weekly, monthly, trimonthly, six-monthly, and yearly averages). As described above, the available data sets, which contain either hourly observations or combined daily and hourly observations, are regarded as a realization of the spatiotemporal RF *X****p*** representing the ambient pollutant, and the space–time dependence of the pollutant is characterized by the joint pdf (1) of the *X****p***. To estimate long-term mean exposure, the available short-time-scale data (hourly and daily) should be upscaled to the larger time scale (monthly, yearly, etc.). Spatiotemporal characteristics at short time scales can be also upscaled to represent long-term exposure characteristics that will be incorporated into the BME framework, as discussed further below. Spatial and/or temporal upscaling has been discussed in several environmental health studies (Choi et al. 2003; Christakos and Hristopulos 1998; Gotway and Young 2002).

In the present study, to estimate air pollution exposures at large time scales, we examined two different upscaling methods: daily data aggregation followed by BME estimation at longer time scales (UM1) and daily BME estimation followed by aggregation at longer time scales (UM2).

#### G-KB

To obtain long-term exposure estimates at the area of interest in terms of the UM1, we first upscaled the data available from the short time scale of observation, (***s***, *t*), to the long-time-scale domain, (***s***, *T*), *T* > *t*; we then generated estimates of the upscaled pollutant exposure. Consider the pollutant RF *X****p***=*X****s****,_t_* with covariance *c_X_* (***p****_i_*, ***p****_j_*) = *c_X_* (***s****_i_*, ***t****_i_*; ***s****_j_*, ***t****_j_*) at the (***s***, *t*) scale. The temporally upscaled RF and the corresponding covariance at the (***s***, *T*) scale are expressed by, respectively,


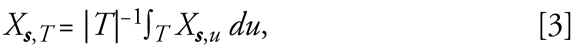


and


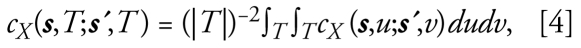


where *T* denotes the time intervals of the upscaled domain within which the original, short-time-scale RF is averaged. Equations 3 and 4 belong to the G-KB of the pollutant. The change of covariance function under a change of support as shown above in spatial analysis is also known as regularization theory (Journel and Huijbregts 1978).

To obtain long-term exposure estimates at the (***s***, *T*) region of interest in terms of the UM2, we first use the BME technique to generate exposure estimates for all locations of interest at the small time scale (***s***, *t*), and then obtain the upscaled estimates from the aggregation of the short-time-scale estimates. In the UM2 context, the G-KB consists of the mean trend and covariance functions at the short-term time scale (***s***, *t*).

#### S-KB

Daily or hourly observations were aggregated into the multitime scale exposure knowledge base. This upscaled uncertain knowledge base of pollutant concentration is represented in terms of a complete probability distribution rather than a single value. As mentioned above, the sampling frequency generally varies among the monitoring stations. Concerning the raw AQS data set used in this study, both daily and hourly PM_10_ observations were available, whereas hourly data were primarily used in the case of ozone. According to the AQS ambient pollutant manual (U.S. EPA 2004), daily observations can be estimated in terms of the arithmetic mean of hourly observations only if the number of these observations is greater than 18 (i.e., ≥ 75% of intended samples); otherwise, we treated them as missing data. Needless to say that, it is not always easy to assure that the long-term exposure information satisfies the 75% criterion above. In fact, the total number of observation days is often less than half the long-term period of interest. Instead of ignoring the scarce observations, as done by the previous methods, in the present study we considered two different avenues toward quantification of the uncertainty of the long-term exposure estimates: (*a*) for the 25–75% sampling period, data pdfs of various shapes were constructed on the basis of the observation histograms; and (*b*) for the < 25% sampling period, uniform distributions were generated on the basis of the arithmetic mean. The ranges of the upscaled exposure data were between 0.25 and 1.75 times the arithmetic mean. If daily and hourly observations coexisted at the same location, the same 24 observed daily values were assigned into the corresponding hours. If daily and hourly observations were collocated, the daily information was considered to be hard data. In this way, BME was able to account for uncertain yet valuable exposure information.

There were 87 (PM_10_) and 77 (ozone) monitoring stations, but the spatial network of pollution monitors never operated fully during the entire 2,922 days of the study period. In fact, the mean (median) number of operating stations in any specific day was 15 (8) stations for PM_10_, and 41 (55) stations for ozone. The maximum (minimum) number of stations per day was 66 (3) for PM_10_ and 69 (7) for ozone, respectively. Moreover, most of the PM_10_ stations obtained observations with a 6-day frequency.

### Spatiotemporal exposure estimation and cross-validation

Daily estimation is the smallest temporal estimation unit in this study. The performance of the BME method in daily PM_10_ and ozone exposure estimation was assessed by cross-validation, using all AQS data available during the study period. Cross-validation allows assessment of the estimation accuracy in different space–time domains and can avoid the potentially biased interpretation of the estimation results induced by purely spatial correlations or purely temporal trends. Therefore, we randomly selected approximately 1,000 observations across space–time to be the estimation points for cross-validation purposes. This selection is based on the objective of achieving a balance between three factors: the desirable size of spatiotemporal clusters, the number of clusters (968 for PM_10_ and 996 for ozone), and the need to reduce the computation burden of the cross-validation of BME estimates at both the daily and the large time scales. The differences of real observations versus BME estimates within each randomly selected spatiotemporal cluster were pooled and assessed across all monitors. For the purpose of comparison, simple kriging with the same spatiotemporal structure for BME method, that is, mean trend and covariance, is also applied to the cross-validation at daily scale.

We also applied the cross-validation of large-scale exposure estimation to assess and compare the predictive accuracy by the two upscaling methods, UM1 and UM2. In UM1, the exposure data were first transformed to the scale of interest, and then the BME technique was applied on the upscaled data, which can be hard or soft, as discussed above, to generate upscaled exposure estimates. In UM2, on the other hand, the daily exposure G-KB and S-KB were processed, as discussed above, and the daily estimates generated by the BME technique, and then the exposure estimates were upscaled to the domain of interest.

In order to produce the long-term exposure estimates, the daily estimates were aggregated as follows:


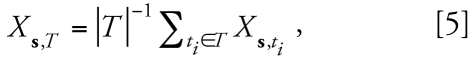


and


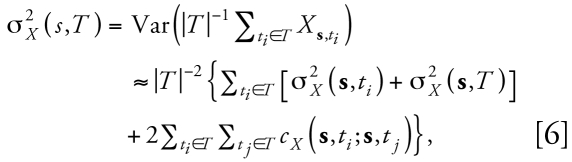


Where σ^2^*_X_* (***s***, *T*) and σ^2^*_X_* (***s***, *t_i_*) are the variances of ***X****_s_*,*_T_* and ***X****_s_*,*_ti_*, respectively, and *c_x_*(***s***, *t_i_*; ***s***, *t_j_*) is the covariance between (***s***, *t_i_*) and (***s***, *t_j_*). Note that in this study the choice of the exposure estimation period (*T*) is different from that in many epidemiologic studies that followed the calendar temporal units. Instead, we define the exposure period in this study as the period that starts at the time of the epidemiologic survey of the participants and retrospectively defines a specified period of interest, making the exposure time window temporally aligned with the timing of collecting health data during the survey.

In the case of multiple-time-scale exposure, we also conducted two additional cross-validation exercises (one for UM1 and one for UM2) to compare the relative performance of the two upscaling methods at large time scales. The idea of cross-validation is to assess estimation accuracy by comparing the exposure estimates with true exposure observations. However, the latter are not directly available at long time scales. To overcome this difficulty, statistical hypothesis tests were implemented to detect if the generated soft exposure data are significantly close to the BME exposure estimates. The “distance” between the pdfs of soft data and the BME estimates was assessed in terms of the relative entropy measure:


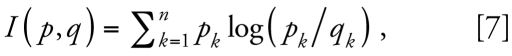


where ***p****_k_* and ***q****_k_* represent the pdfs of the exposure observations and the BME estimates, respectively. The goodness-of-fit test is usually applied to verify if the two pdfs come from the same random variable. Chi-square distribution with *n* − 1 degrees of freedom can be used in the relative entropy measure tests (Bedford and Cooke 2001). The significance criterion for the tests was set as 95%. Cross-validation for the UM1 and UM2 methods at long time scales was performed at the same temporally-referenced points as in the case for the cross-validation of daily BME estimation.

Finally, we applied both UM1 and UM2 to estimate PM_10_ and ozone exposures at multiple time scales for all the residential locations of the HEAPL study. The correlation coefficients for each BME estimate at different time scales were computed for the UM1 and UM2 methods and compared accordingly. We also examined the distribution of the differences between the UM1 and UM2 estimates at different time scales.

## Numerical Results and Plots

Table 1 presents the cross-validation results for the daily PM_10_ and ozone data by BME and kriging methods. The exposure estimation error at each test point is defined as error = estimate − observation. In general, both the error mean and median are close to zero, so the error distribution is symmetric around zero. To compare the average exposures at multiple time scales from real observations versus the BME estimates, Tables 2 and 3 show the results from UM1 and UM2. Table 2 summarizes simple statistics of the estimation errors given by UM1 and UM2 for both PM_10_ and ozone, and Table 3, results of corresponding comparison on relative entropy at each indicated time scale, showing the percentage of the spatiotemporal estimates that passed the chi-square tests with the null hypothesis: the two pdfs (data and estimates) are the same.

**Figure 3:**
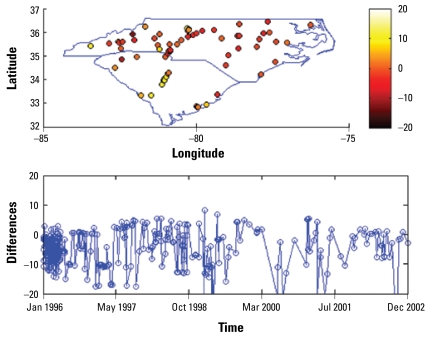
Spatiotemporal yearly PM_10_ estimation errors (estimated − observed) by UM1.

**Figure 4:**
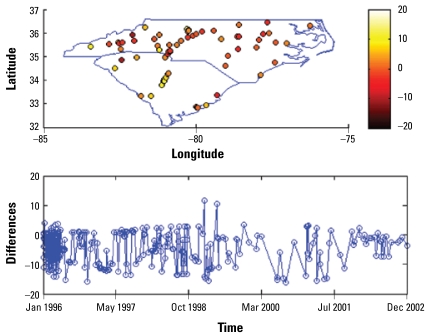
Spatiotemporal yearly PM_10_ estimation errors (estimated − observed) by UM2.

**Figure 5:**
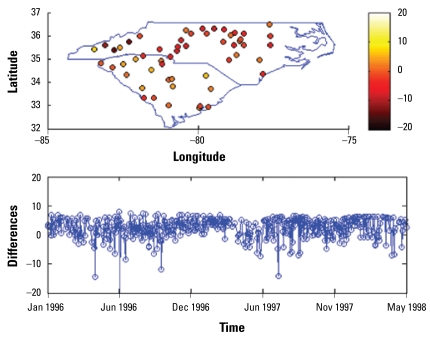
Spatiotemporal yearly ozone estimation errors (estimated − observed) by UM1.

**Figure 6:**
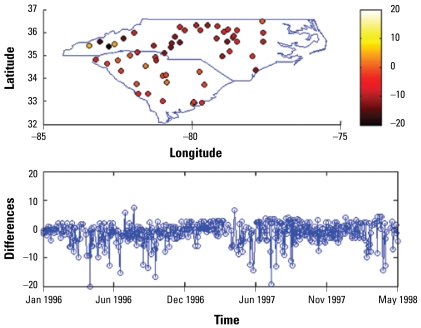
Spatiotemporal yearly ozone estimation errors (estimated − observed) by UM2.

Figures 3–6 show the spatial and temporal distributions of the average estimation errors of the yearly exposure estimates obtained by UM1 and UM2. Figures 3 and 4 show the PM_10_ estimation performance by means of UM1 and UM2, respectively. Similarly, Figures 5 and 6 show the average error distributions of ozone estimation obtained by UM1 and UM2, respectively.

**Figure 7:**
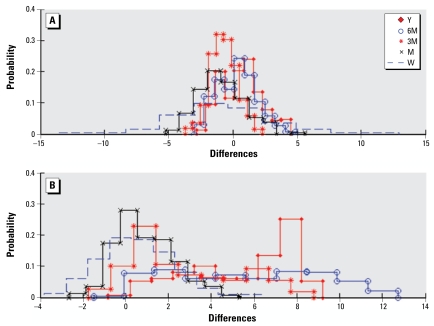
Abbreviations: M, 1 month; 3M, 3 months; 6M, 6 months; W, 1 week; Y, 1 year. Distribution of differences between the UM1 and UM2 exposure estimates for PM_10_ (*A*) and ozone (*B*) at multiple time scales.

Table 4 presents the summary statistics for the calculated differences in UM1–UM2 that were tabulated, respectively, for PM_10_ and ozone exposure at each indicated time scale. Figure 7 shows the histograms of these differences for both methods. Table 5 shows the correlation coefficients between the PM_10_ and ozone exposure estimates obtained by UM1 and UM2 within each temporal scale at the study residences.

## Discussion

Scale laws and scaling behaviors at multiple time scales are encountered in many human exposure scenarios, although very often such laws are found in an empirical way, because of the lack of fundamental theories allowing us to understand them from fundamental principles (Christakos and Hristopulos 1998). In the case of chronic diseases, the arithmetic mean of long-term (large time scale) participant exposure rather than the on-site exposure is often considered as the appropriate indicator (AckermannLiebrich et al. 1997; Pope et al. 2002). For regulatory purposes, the National Ambient Air Quality Standards (NAAQS) proposed by U.S. EPA are also based on the arithmetic mean exposure at different time scales, which range from hourly to annual exposure (U.S. EPA 2006). Many studies have focused on long-term arithmetic mean exposure estimates based on small time scale (short-term) observations and assuming lognormal RF to model exposure distributions (Clayton et al. 1999; Wallace and Williams 2005). In general, these studies do not consider important spatiotemporal dependencies between short-term observations and cross-dependencies between short- and long-term exposures.

In this article, we present two upscaling methods and compare them for the estimation of arithmetic average exposures within the different temporal scales. As described in the introductory remarks, previous data analyses often did not consider the uncertainty of the exposure analysis (e.g., by purely spatial or purely temporal analysis or linear assumptions). For the upscaling problem considered here, this uncertainty may be a significant factor in many human exposure situations; for example, in the case of PM_10_ data with a distinct trend and a large number of missing values (because most monitors only record every 6 days), the estimation of the long-term exposure averages can be seriously biased.

As mentioned above, the AQS manual suggests that when there is a large number of missing data the accuracy of the upscaled exposure is in doubt, in which case the rest of the observed information should be ignored. Accordingly, mainstream statistics and geostatistics techniques usually consider incomplete information (qualitative knowledge, uncertain secondary records, etc.) as missing data to avoid potentially misleading estimation results. On the other hand, the BME method used in this study has the significant feature that it is able to rigorously incorporate uncertain information of various kinds and different scales with the minimum number of theoretical assumptions. In other words, the BME method can always express incomplete information in terms of soft site-specific data that can take the form, for example, of probability functions with arbitrary shapes. In addition, BME can incorporate empirical relations and charts as well as core knowledge in the form of epidemiologic laws and scientific human exposure models, whenever available (Christakos et al. 2005). Because of the abundance of missing data, the uncertain (soft) information is available for both PM_10_ and ozone BME predictions at all concerned time scales in this study. Table 1 provides the cross-validation results of daily PM_10_ and ozone estimations by BME and kriging methods and shows that the estimation error distribution of the results of BME method is more condensed and symmetric around zero. The improvement of the estimation accuracy by integrating soft data in BME method is more significant as the amount of missing data is greater, such as the case of PM_10_.

Concerning the comparison of the accuracies of the two upscaling approaches: based on the cross-validation results (Table 2 and 3), the UM2 is generally better than UM1 in terms of smaller mean and median errors and higher success rates of passing the chi-square tests of uncertain information. Table 2 shows that the standard deviation of the differences between observations and estimates decreases as the estimation time scale increases (for both PM_10_ and ozone cases). This is because the aggregated hard and soft data (which emerge as the time scale increases) can lead to a reduction of the estimation uncertainty and provide more informative exposure estimates. In the case of the PM_10_ data set, for example, during the study period of interest about 5,000 more spatiotemporal data are compiled in the yearly database than in the weekly database. The UM1 and UM2 methods generally underestimate the real PM_10_ levels. The preferential sampling of high PM_10_ values can partially contribute to the biased estimations. Also, some extreme high values in PM_10_ data set can also bias the estimations at the process of normal score transform.

Geostatistical techniques generate estimates in terms of spatial and temporal interpolation schemes, which rely on linearity and normality assumptions and tend to generate rather smooth PM_10_ estimates. On the other hand, the UM1 and UM2 use the BME approach that does not make any linearity or normality assumption (nonlinear estimators and non-Gaussian distributions are automatically incorporated) and can rigorously process uncertain yet valuable data sources (e.g., soft data of various forms), thus providing more informative estimates than the geostatistical techniques.

**Figure 8:**
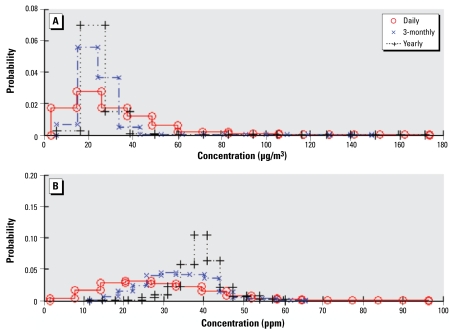
Distribution of means of upscaled PM_10_ (*A*) and ozone (*B*) data.

In the case of highly uncertain data, some extremely high observations may not be completely reproduced. Even though both upscaling methods underestimate the actual PM_10_ exposures, the UM2 performs better than UM1 yielding lower estimation errors. In the case of ozone, the performance of UM1 is significantly different than that of UM2. UM1 tends to overestimate the long-term exposure level, and the situation worsens as the estimation scale becomes larger. Remarkably, the UM2 exposure estimates are not biased, whereas the biased UM1 estimation is likely due to the aggregation of the ozone data set. Because of the seasonal ozone pattern, the distribution of daily ozone data during the study period is positively skewed, ranging from 0 to 70 ppb. However, when temporal aggregation was applied, the mean of the upscaling data generally raised to the annual mean level at each spatial location, which may distort the original spatiotemporal ozone pattern at the smaller time scales. As shown in Figure 8, the distribution of the mean of the aggregated ozone data varies significantly by the degree of upscaling, which is not the case of the PM_10_ estimation. Moreover, UM2 does not depend on any distorted upscaled data, so more accurate results are obtained. Despite the significant changes in data structure during aggregation, the rigorous consideration of data uncertainty by BME alleviates such effects to produce better quality estimates (Table 2). Table 3 shows that the estimates are generally superior for ozone than for PM_10_. This is because most PM_10_ monitors performed air sampling every 6 days, in which case the resulting upscaled long-term exposure is less informative of the exposure situation, especially at the short time scales. Therefore, the shorter the upscaling period considered (e.g., weekly), the more noninformative uncertain data are compared with estimations.

Figures 3–6 plot the spatial and temporal distributions of the UM1 and UM2 results. In the PM_10_ case, the spatial and temporal patterns of the error distributions obtained by the UM1 and UM2 methods are very similar. These plots offer a better understanding of the performance of the proposed approach in space–time. The conclusion drawn from Table 2 concerning long-term PM_10_ underestimation is also illustrated by the temporal error distributions plotted in figures 3–6. In the case of ozone estimation, these figures also depict a similar conclusion drawn from Table 2 (i.e., UM1 tends to overestimate the long-term ozone levels). It is noteworthy that spatial locations where the estimates exhibit higher discrepancies from the data values (for both PM_10_ and ozone) are mostly close to either the boundary between regions of considerable data availability and data scarcity or the metropolitan area where the high variability of PM pollutants and ozone generated from traffic or local industrial emissions may be present.

The mean and median of the differences between the UM1 and UM2 estimates specific to the residential locations in HEAPL at multiple timescales are mostly close to each other and not much departing from zero for both pollutants (Table 4), except in the case of long-term ozone estimation. The estimates obtained by UM1 are biased, so UM1 generates higher ozone levels than UM2, which can be seen more clearly from the histograms at the bottom of Figure 7. In general, the UM1 and UM2 estimates should get closer to each other as the time scale increases under the condition of the unbiased aggregated data provided. As the time scale increases, the number of daily values increases for both upscaling methods (i.e., more data become available for aggregation purposes in the case of UM1, whereas more estimates are generated for integration purposes in the case of UM2). As a consequence, based on the central limit theorem, the exposure mean is optimally calculated at the longer time scale by both upscaling methods (Figure 8), as shown in the case of PM_10_ estimation. However, the exposure estimation accuracy may also decrease if the data uncertainty resulting from the large proportion of missing data or biased aggregated data is large, which is the case of ozone estimation at long time scales. Thus, the mean and median of the differences between the estimation results by UM1 and UM2 can slightly increase with time scale.

In this study, numerical analysis showed that UM2 generally performs slightly better than UM1 in terms of accuracy. UM2 can also be preferable in theory. Instead of aggregating the data and spatiotemporal dependence at small scales, BME analysis incorporates G-KB and S-KB, including detailed local spatiotemporal associations and the original short-term observations. In UM1, on the other hand, both general and specific knowledge are upscaled, so the BME estimation uses the more uncertain information. However, despite the better performance of UM2, in practice the UM1 may be sometimes preferable because of its efficiency. The difference of computation burden between the two approaches increases substantially as the estimation time scale increases. As the exposure estimation at residential locations shows, the UM1 can generate biased estimates in the case of ozone but not in the case of PM_10_. This suggests the criterion for the selection of UM1 and UM2 in the long-term exposure estimations. UM1 is preferable as long as the aggregation process does not change the original data structure, that is, mean trend and variance/covariance of the data. In such cases, the loss of information during the data aggregation in UM1 can be neglected compared with the increase of time for the estimations by UM2; otherwise, UM2 is preferable. In this study, because of the strong seasonal ozone trend, an aggregation period exceeding 3 months can distort the spatiotemporal data structure.

## Conclusions

To estimate residential levels of exposure to ambient air pollution in a community-based study, in this article we presented and compared two BME-based temporal upscaling methods (UM1: data aggregation followed by BME estimation; and UM2: BME estimation followed by aggregation). BME’s flexibility allowed the assimilation of G-KB and S-KB of different formats; for example, BME exposure analysis can process scarce and uncertain data sets in a probabilistic way, instead of neglecting them, as is the case with most existing quantitative exposure methods. In the context of residential long-term exposure estimation, we showed that the UM1 and UM2 methods produce accurate space–time estimates. By means of cross-validation tests the relative performance of the two upscaling methods was studied in different time scales. We found UM2 to be generally better than UM1, in the sense that the UM2 estimates were unbiased, the differences between the UM2 estimates and the true long-term exposures were smaller, and the UM2 exhibited better test-passing rates than UM1. On the other hand, the UM1 can perform reasonably well when the aggregation process does not alter the spatiotemporal structure of the original data set.

## Figures and Tables

**Table 1:** BME and kriging cross-validation results (statistics of estimation errors for daily estimates).

Measure	No. of estimation points	Method	Mean	SD	Median
PM_10_ (μg/m^3^)	968	BME	−0.7144	8.1919	0.2316
		Kriging	−2.6136	13.2484	−0.0285
Ozone (ppm)	996	BME	0.1768	6.8450	0.3427
		Kriging	0.4831	7.0352	0.6234

**Table 2:** Summary statistics of the cross-validation results for the BME long-term exposure estimates derived by UM1 and UM2.

	UM1			UM2		
Time period	Mean	Median	SD	Mean	Median	SD
PM_10_						
1 Week	−2.8944	−3.2590	7.8800	−1.9057	−1.1636	7.9308
1 Month	−3.1889	−2.0855	7.2113	−2.4693	−1.2410	7.1029
3 Months	−3.2260	−2.1502	6.9366	−2.5038	−1.0816	7.0235
6 Months	−3.2732	−2.2481	7.0015	−2.2868	−0.7581	6.8586
1 Year	−3.0810	−3.2590	6.5565	−2.2919	−1.3173	6.4123
Ozone						
1 Week	1.9795	1.9350	4.9122	2.3559	2.4536	5.1754
1 Month	1.6399	1.6287	4.2774	1.5039	1.8269	4.2932
3 Months	2.1265	2.4236	4.1578	0.7233	1.1856	4.3105
6 Months	2.4861	2.8128	4.0799	−0.5237	−0.2164	4.3031
1 Year	2.4537	2.9891	3.7602	−1.3781	−0.7395	3.8705

**Table 3:** Percentage of successful cross-validation results of long-term exposure estimation.

Time period	PM_10_		Ozone	
	UM1 (%)	UM2 (%)	UM1 (%)	UM2 (%)
1 Week	10.16	11.18	74.60	55.89
1 Month	71.49	74.54	54.15	61.71
3 Months	76.11	77.38	64.87	95.78
6 Months	77.66	79.18	81.64	95.41
1 Year	73.47	74.69	82.90	95.96

**Table 4:** Summary statistics of the differences between UM1 and UM2 estimates of PM_10_ and ozone given for all residential locations.

		Statistics of differences between UM1 and UM2 estimates				
Pollutant	Measure	Weekly	Monthly	3-Monthly	6-Monthly	Yearly
PM_10_	Mean	−0.9901	−0.8948	−0.4612	0.1714	0.6001
	SD	3.9488	1.9799	1.4735	1.7279	1.9247
	Median	−0.9381	−1.0235	−0.6006	0.0975	0.4830
Ozone	Mean	0.2874	0.5971	1.9185	4.5729	5.5210
	SD	1.9585	1.6888	2.5553	3.5355	2.9941
	Median	0.2288	0.4591	1.2509	4.3353	6.2256

**Table 5:** Correlation coefficients among multitemporal-scale exposure estimations for PM_10_ and ozone.

		Ozone					
PM_10_	Time period	1 Day	1 Week	1 Month	3 Months	6 Months	1 Year
UM1	1 Day	1	0.6981	0.6286	0.4065	0.0881	0.0476*
	1 Week	0.4966	1	0.8608	0.6372	0.2399	0.0686
	1 Month	0.3595	0.5608	1	0.8308	0.3959	0.0887
	3 Months	0.2768	0.3954	0.7039	1	0.7195	0.1953
	6 Months	0.1141	0.1863	0.3302	0.6422	1	0.5693
	1 Year	0.1249	0.1627	0.2753	0.3015	0.6205	1
UM2	1 Day	1	0.7972	0.6744	0.4143	0.0168*	0.1843
	1 Week	0.6303	1	0.8543	0.5618	0.0733	0.1982
	1 Month	0.425	0.6495	1	0.7854	0.2485	0.2476
	3 Months	0.3179	0.4487	0.7423	1	0.7233	0.3731
	6 Months	0.1422	0.1899	0.34	0.7431	1	0.551
	1 Year	0.1288	0.166	0.3061	0.4609	0.6857	1

* *p* > 0.05.
